# Performance of plasma Aβ42/40, measured using a fully automated immunoassay, across a broad patient population in identifying amyloid status

**DOI:** 10.1186/s13195-023-01296-5

**Published:** 2023-09-04

**Authors:** Shogyoku Bun, Daisuke Ito, Toshiki Tezuka, Masahito Kubota, Ryo Ueda, Keisuke Takahata, Sho Moriguchi, Shin Kurose, Yuki Momota, Natsumi Suzuki, Ayaka Morimoto, Yuka Hoshino, Morinobu Seki, Yu Mimura, Ryo Shikimoto, Yasuharu Yamamoto, Takayuki Hoshino, Yoshiaki Sato, Hajime Tabuchi, Masaru Mimura

**Affiliations:** 1 Department of Neuropsychiatry, Keio University School of Medicine, 35 Shinanomachi, Shinjuku-Ku, Tokyo, 160-8582 Japan; 2 Memory Center, Keio University School of Medicine, Tokyo, Japan; 3 Department of Physiology, Keio University School of Medicine, Tokyo, Japan; 4 Department of Neurology, Keio University School of Medicine, Tokyo, Japan; 5 Office of Radiation Technology, Keio University Hospital, Tokyo, Japan; 6 Department of Functional Brain Imaging Research, National Institute of Radiological Sciences, National Institutes for Quantum and Radiological Science and Technology, Chiba, Japan; 7 Graduate School of Media and Governance, Keio University, Kanagawa, Japan; 8 Eisai-Keio Innovation Laboratory for Dementia, Human Biology Integration Foundation, Eisai Co., Ltd, Tokyo, Japan

**Keywords:** Amyloid β, Plasma Aβ42/40, Alzheimer’s disease, Amyloid positron emission tomography, Centiloid

## Abstract

**Background:**

Plasma biomarkers have emerged as promising screening tools for Alzheimer’s disease (AD) because of their potential to detect amyloid β (Aβ) accumulation in the brain. One such candidate is the plasma Aβ42/40 ratio (Aβ42/40). Unlike previous research that used traditional immunoassay, recent studies that measured plasma Aβ42/40 using fully automated platforms reported promising results. However, its utility should be confirmed using a broader patient population, focusing on the potential for early detection.

**Methods:**

We recruited 174 participants, including healthy controls (HC) and patients with clinical diagnoses of AD, frontotemporal lobar degeneration, dementia with Lewy bodies/Parkinson’s disease, mild cognitive impairment (MCI), and others, from a university memory clinic. We examined the performance of plasma Aβ42/40, measured using the fully automated high-sensitivity chemiluminescence enzyme (HISCL) immunoassay, in detecting amyloid-positron emission tomography (PET)-derived Aβ pathology. We also compared its performance with that of Simoa-based plasma phosphorylated tau at residue 181 (p-tau181), glial fibrillary acidic protein (GFAP), and neurofilament light (NfL).

**Results:**

Using the best cut-off derived from the Youden Index, plasma Aβ42/40 yielded an area under the receiver operating characteristic curve (AUC) of 0.949 in distinguishing visually assessed ^18^F-Florbetaben amyloid PET positivity. The plasma Aβ42/40 had a significantly superior AUC than p-tau181, GFAP, and NfL in the 167 participants with measurements for all four biomarkers. Next, we analyzed 99 participants, including only the HC and those with MCI, and discovered that plasma Aβ42/40 outperformed the other plasma biomarkers, suggesting its ability to detect early amyloid accumulation. Using the Centiloid scale (CL), Spearman’s rank correlation coefficient between plasma Aβ42/40 and CL was -0.767. Among the 15 participants falling within the CL values indicative of potential future amyloid accumulation (CL between 13.5 and 35.7), plasma Aβ42/40 categorized 61.5% (8/13) as Aβ-positive, whereas visual assessment of amyloid PET identified 20% (3/15) as positive.

**Conclusion:**

Plasma Aβ42/40 measured using the fully automated HISCL platform showed excellent performance in identifying Aβ accumulation in the brain in a well-characterized cohort. This equipment may be useful for screening amyloid pathology because it has the potential to detect early amyloid pathology and is readily applied in clinical settings.

**Supplementary Information:**

The online version contains supplementary material available at 10.1186/s13195-023-01296-5.

## Background

In current clinical practice, diagnosing Alzheimer’s disease (AD), the most common cause of dementia worldwide [1], is essentially based on clinical findings with or without the aid of brain imaging, such as magnetic resonance imaging (MRI). However, the accuracy of the clinical diagnosis may be inadequate [2] when referenced to the gold standard neuropathological findings, including amyloid β (Aβ) plaques and intracellular accumulation of the hyperphosphorylated protein tau as neurofibrillary tangles in the brain [3]. The current National Institute on Aging-Alzheimer’s Association research framework [4] may bridge the gap between clinical and neuropathological diagnoses by requiring Aβ positivity to diagnose AD. However, the use of amyloid positron emission tomography (PET) imaging or cerebrospinal fluid (CSF) Aβ measurements, which can identify Aβ deposition in the brain, are limited in regular clinical settings owing to high cost, advanced facility requirements, or procedural invasiveness. Thus, accessible, scalable, and reliable diagnostic tests for screening Aβ deposition in the brain are required.

Recently, plasma biomarkers have emerged as promising screening tools for AD. One such candidate is the plasma Aβ42/40 ratio (Aβ42/40). Plasma Aβ measurements were initially considered impractical [5]; however, recent studies have shown more positive results. For example, a fully automated Elecsys immunoassay method reported areas under the curve (AUC) of 0.83–0.87 [6]. Another fully automated Aβ42/40 measurement using high-sensitivity chemiluminescence enzyme (HISCL) immunoassay platform [7, 8] yielded AUCs of 0.87–0.94. The measurement by the HISCL platform was validated using samples from the Elenbestat Phase 3 global multicenter clinical trials, which consisted of racially diverse participants clinically diagnosed with mild AD or mild cognitive impairment (MCI) due to AD. One of the advantages of using these fully automated immunoassays is that they are already on the market and can be widely applied in regular clinical settings if their ability and robustness are validated with a broader population. Therefore, we aimed to examine the performance of plasma Aβ42/40, measured using the fully automated HISCL immunoassay, in detecting amyloid-PET-derived Aβ pathology in a well-characterized memory clinic cohort [9]. This allows us to verify its practicality in a more diverse patient population, expanding beyond the original report’s inclusion of only MCI and mild AD in clinical trials. Furthermore, given the importance of early detection of AD, we sought to evaluate its usefulness in a subpopulation consisting sorely of healthy controls and MCI. Additionally, we aimed to compare its performance against that of other potential plasma biomarkers, namely Simoa-based plasma phosphorylated tau at residue 181 (p-tau181), glial fibrillary acidic protein (GFAP), and neurofilament light (NfL) [10].

## Methods

### Participants

This is a cross-sectional study that recruited patients from the Memory Clinic at Keio University Hospital and healthy controls from a patient recruitment agency (3H Medi Solution Inc., Tokyo, Japan). Patients’ clinical diagnoses included AD, frontotemporal lobar degeneration (FTLD, including progressive supranuclear palsy, corticobasal syndrome, behavioral-variant frontotemporal dementia, or primary progressive aphasia), dementia with Lewy bodies/Parkinson’s disease (DLB/PD), MCI, and other disorders, including traumatic brain injury (TBI) and mental disorders, such as depression or delusional disorder. The recruitment period was from July 2018 to December 2022, and all diagnostic criteria were followed [11–17].

Inclusion criteria for enrollment were as follows:

All participants must be between 40–85 years with education years ≥ 12. HCs must have a Mini-Mental State Examination (MMSE) [18] score ≥ 24, Clinical Dementia Rating (CDR) [19] = 0, Wechsler Memory Scale Logical Memory test II score ≥ 5 or ≥ 9 depending on education years (9–15 or ≥ 16), and Geriatric Depression Scale [20] score < 6. Patients with AD must have an MMSE score ≤ 23 and CDR = 0.5 or 1. Patients with MCI must have an MMSE score ≥ 24, CDR = 0.5 with memory domain ≥ 0.5, and Wechsler Memory Scale Logical Memory test II score ≤ 9 or ≤ 11, depending on education years.

Exclusion criteria for any dementia were as follows: concurrent diagnosis of other neurodegenerative or neurological diseases than the ones listed above, history of major depressive disorder or bipolar disorder within a year before enrollment, history of any substance-related or addictive disorder within 2 years before enrollment, or history of schizophrenia diagnosis at any time.

A board-certified neurologist performed a comprehensive medical and neurological workup, including neurological examination, routine blood work, complete blood count, blood chemistry, thyroid function tests, vitamin B12/folate measurements, 3-Tesla MRI, and amyloid PET scanning, for all participants.

### MRI

Three-dimensional T1-weighted imaging (3D BRAVO, repetition time = 6.8 ms, echo time = 3.0 ms, field of view = 23.0 mm, voxel size = 0.9 × 0.9 × 1.0 mm, and flip angle = 8˚) was performed using a Discovery MR750 3.0 T scanner (GE Healthcare, USA) at Keio University Hospital.

### Amyloid PET imaging

A 20-min static scan was performed 90 min after the intravenous infusion of 300 MBq ± 10% ^18^F-Florbetaben [21, 22], using a PET/computed tomography (CT) system (Siemens Biograph mCT or Siemens Biograph mCT flow, Munich, Germany). ^18^F-Florbetaben was manufactured according to good manufacturing practice at Keio University Hospital with an automated synthesizer (Synthera V2; IBA, Louvain-la-Neuve, Belgium). The acquired PET data were reconstructed by an ordered subsets expectation maximization algorithm (4 iterations, 24 subsets), using a matrix size of 200 × 200, full width at half maximum (FWHM) Gaussian post-reconstruction filtering of 3 mm, and scatter correction. CT (tube voltage: 120 kVp; tube current: 50 mAs, 0.5 s per rotation; slice thickness: 2 mm) was performed for attenuation correction and anatomic registration. A neuroradiologist who completed a required training assessed the reconstructed images visually as Aβ-positive or Aβ-negative [23]. Briefly, in the visual assessment, readers used axial PET slices to compare the signal intensity between the gray and white matter at the lateral temporal, frontal, and parietal lobes, and posterior cingulate cortex/precuneus and scored using the regional cortical tracer uptake (RCTU) scoring system. When tracer uptake in the gray matter was equal to or higher than that in the adjacent white matter, the RCTU score was two or three, meaning positive tracer uptake, whereas a score of one meant no tracer uptake. Subsequently, each RCTU score of the four brain regions was aggregated into the brain amyloid plaque load score, and Aβ positivity was determined. If one or more RCTU scores were more than one, Aβ was determined to be positive [21].

### The Centiloid (CL) scale [24] calculation

We used “Amyquant,” [25] a recently developed standalone software for semi-automatic quantitative analyses of brain amyloid PET, to calculate CL. It enables reliable calculation of the global CL and amyloid accumulations (quantified as standard uptake value ratio, SUVR) in the five important regions (including the posterior cingulate cortex and precuneus, frontal cortex, temporal cortex, parietal cortex, and striatum). Currently, it applies to the five amyloid PET tracers, including ^18^F‐florbetaben. We adopted the whole cerebellum as a reference region [26]. The accuracy of the calculated CL values was validated by comparing the results to those published on the Global Alzheimer's Association Interactive Network website (https://www.gaain.org/centiloid-project).

### Plasma biomarker measurement

Fasting venous blood samples were collected in ethylenediaminetetraacetic acid (EDTA)-2K-containing tubes (Becton, Dickinson Vacutainer™ Plastic Blood Collection Tubes with K2EDTA) and placed on ice. The samples were centrifuged (1200 *g* for 10 min) within 2 h of the blood draw, followed by further centrifugation in different tubes (2800 *g* for 10 min), which resulted in platelet-free plasma within 30 min. They were aliquoted into polypropylene tubes (Thermo Scientific™ Matrix™ 2D Barcode tube 1.0 mL) and stored at − 80°C until the assay.

Plasma p-tau181, NfL, and GFAP were measured using the commercial Quanterix® assay (Simoa® p-Tau181 Advantage Kit, Simoa® NF‐light Kit, or Simoa® GFAP Discovery Kit) on an HD‐1 analyzer or SR–X, in accordance with the respective manufacturer’s instructions (Quanterix, Billerica, MA, USA). Plasma Aβ40 and Aβ42 levels were measured using the automated HISCL platform (Sysmex HISCL-5000, Japan) as described in the reference study [7].

### CSF Aβ measurement

We obtained CSF from a subset of participants who consented to undergo a lumbar puncture procedure. Fasting CSF samples were collected in 15 mL ProteoSave tubes (Sumitomo Bakelite Co., Ltd.) and placed on ice. The samples were centrifuged (70 g for 10 min) within 2 h of sample collection. They were aliquoted into polypropylene tubes (Thermo Scientific™ Matrix™ 2D Barcode tube 1.0 mL) and stored at − 80°C until the assay. The levels of Aβ1–40 and Aβ1–42 were examined by ELISA on SpectraMax M5e plate reader (Molecular Devices LLC, Sunnyvale CA) according to the manufacturer’s protocols (Wako, Japan). A 100 μl of diluted CSF (1:25 dilution) from each sample was assayed, with an equal volume of standard solution as an internal control.

### Apolipoprotein E (APOE) status

Genotyping for the APOE alleles (rs429358 and rs7412) was performed in MCBI (Ibaraki, Japan) to determine the three major isoforms (APOE ε2, APOE ε3, and APOE ε4). Briefly, genomic deoxyribonucleic acid (DNA) was extracted from 0.2 mL whole blood using the Magnetic Nanoparticles DNA Extraction kit (EZ1 DNA Blood 200 μL Kit). APOE genotyping was performed via real-time polymerase chain reaction using the TaqMan probe on a CFX 96 deep well Real-Time polymerase chain reaction system (Bio-Rad, Richmond, CA), following a slightly modified methodology from that described in a previous report [27].

### Cognitive assessment

Cognitive function was assessed using CDR, MMSE, and the Alzheimer’s Disease Assessment Scale, Cognitive Behavior Section (ADAS-cog) [28].

### Statistical analysis

#### Demographics and plasma biomarker values stratified by amyloid PET results

Differences in demographics and plasma biomarker values between amyloid PET-negative and -positive participants were explored using the Mann–Whitney U test for continuous variables and the Chi-square test for categorical variables.

#### Receiver operating characteristic (ROC) analysis

We examined the ability of the Aβ42/40 ratio to predict amyloid positivity determined using visual assessment of amyloid PET by plotting the ROC curve. The AUC was calculated, and the best cut-off value for the plasma Aβ42/40 ratio was determined according to the maximized Youden Index. Subsequently, we investigated whether incorporating additional variables into Aβ42/40 would improve the AUC by employing predicted probability derived from logistic regression analyses. We compared three models: 1) adding APOE4 only, 2) adding APOE4, sex, and age, and 3) adding variables selected among the following set using a forward stepwise logistic regression analysis: the four plasma biomarkers, APOE4, sex, age, MMSE score, and clinical diagnoses (parsimonious model). The comparisons of ROC curves were based on the method used by DeLong et al. [29]. Further, we compared the predictive ability of the four plasma biomarkers by calculating AUC for each biomarker using the 167 participants who had measurements for all four plasma biomarkers. Lastly, given the importance of early detection of amyloid for early intervention, we repeated the same comparison analyses of the four biomarkers in 99 participants who were HCs or those who had MCI and 57 HCs only.

#### Association between plasma Aβ42/40 and CL

We analyzed the association between plasma Aβ42/40 and CL. We employed two CL cut-offs [30, 31]: the lower and higher cut-offs for initial and established amyloid pathology. While established amyloid pathology generally refers to significant amyloid accumulation that can differentiate between clinical AD and healthy controls, initial amyloid pathology indicates subtle amyloid accumulation defined by CSF Aβ levels [31] or deviation from young healthy controls in amyloid PET SUVR [30]. As reported in recent studies [30, 31], a “gray zone”, defined by the CL values falling between the two cut-offs, may indicate early amyloid accumulation. Moreover, Bullich et al. [30] demonstrated that participants within the “gray zone” exhibited more amyloid accumulation in subsequent years compared to amyloid-negative participants. Considering that visual assessment of amyloid PET might not always be sensitive to early amyloid accumulation [30], the “gray zone” may serve as a useful tool in this context. Aligning with previous research [30] using ^18^F-Florbetaben, cut-offs for the initial and established amyloid pathology were set at 13.5 and 35.7, respectively.

#### Correlations between plasma biomarkers, amyloid PET, and cognitive test results

Using Spearman’s rank correlation test, we explored the correlations between the four plasma biomarkers, CL, cognitive test results, and age. The correlations between plasma Aβ42/40 and amyloid SUVR in the five brain regions were also examined.

#### Correlation between plasma and CSF Aβ42/40

Lastly, using data from a subset of participants with available CSF samples, we examined the correlation between plasma and CSF Aβ42/40.

All statistical analyses were conducted using SPSS ver28.0.1.1 and GraphPad Prism9.

## Results

### Demographics

Among the 210 participants enrolled in this study, amyloid PET results were available for 198, of which 197 had one or more plasma biomarker measurements (197 participants had p-tau181 and NfL, 172 had GFAP, and 174 had Aβ42/40 measurements). In addition, outlier biomarker values, defined as measurements greater than three standardized deviations from the mean (including three measurements for p-tau181, two for NfL, three for GFAP, and none for Aβ42/40), were excluded from each analysis.

Table 1 presents the demographics, cognitive outcomes, and plasma biomarker values of the 174 participants who underwent plasma Aβ42/40 measurements, stratified by amyloid PET status.

**Table 1:** Demographics, cognitive outcomes, and plasma biomarker values

	*Amyloid PET*		
	*Positive*	*Negative*	*p-value*
	*Median (IQR) or N (%)*		
*N* = *174*	65 (37.4%)	109 (62.6%)	
Age	75 (70–80)	69 (60–75)	< 0.001
Sex, No. of men/women	33/32 (50.8%)	53/56 (48.6%)	0.784
APOE ɛ4, No. of positive participants (%)	36/29 (55.4%)	25/84 (22.9%)	< 0.001
MMSE score^a^	24 (21–27.5)	29(26–30)	< 0.001
ADAS-Cog score^b^	11.2 (6.3–20.0)	4.7 (3.1–7.5)	< 0.001
Aβ42/40 (× 10^–2^)	8.49 (8.23–8.87)	10.9 (10.2–11.3)	< 0.001
p-tau181 (pg/ml)^c^	3.47 (2.58–4.31)	1.73 (1.32–2.33)	< 0.001
GFAP (pg/ml)^d^	365 (281–542)	213 (162–283)	< 0.001
NfL (pg/ml)^e^	25.4 (19.6–32.8)	18.8 (15.1–25.8)	< 0.001
Clinical diagnosis			< 0.001
HC	10 (15.4%)	48 (44.0%)	
MCI	23 (35.4%)	20 (18.3%)	
AD	27 (41.5%)	5 (4.6%)	
FTLD	4 (6.2%)	20 (18.3%)	
DLB/PD	1 (1.5%)	4 (3.7%)	
Others	0 (0%)	12 (11.0%)	

Positive amyloid PET was determined via visual reading

*Abbreviations*: *IQR* interquartile range, *APOE* apolipoprotein E, *MMSE* Mini-Mental State Examination, *ADAS-Cog* Alzheimer's Disease Assessment Scale Cognitive Behavior Section, *Aβ42/40* amyloid β 42/40 ratio, *p-tau181* tau protein phosphorylated at residue 181, *NfL* neurofilamsent light, *GFAP* glial fibrillary acidic protein, *HC* healthy control, *MCI* mild cognitive impairment, *AD* Alzheimer's disease, *FTLD* frontotemporal lobar degeneration, *DLB/PD* dementia with Lewy bodies/Parkinson's disease

^a^ *N* = 65/106

^b^ *N* = 64/106

^c^ *N* = 64/108

^d^ *N* = 63/106

^e^ *N* = 65/108 for Aβ PET-positive and -negative participants, respectively

One participant was deemed amyloid-negative neuropathologically at autopsy, as the patient passed away before amyloid PET imaging. Considerable differences were observed in all parameters except for sex. In each of the amyloid PET-positive and -negative groups, the median age was 75 and 69, with nearly an equal distribution of sex in both groups. The percentage of APOE ɛ4 positivity was 55,4% and 22.9%, while the median MMSE and ADAS-Cog scores were 24 and 29, and 11.2 and 4.7, respectively. Plasma biomarker values were 0.0849 and 0.109 for Aβ42/40, 3.47 and 1.73 for p-tau181, 365 and 213 for GFAP, and 25.4 and 18.8 for NfL, respectively. Furthermore, there were significant differences in clinical diagnosis between the two groups. AD was the most frequent diagnosis among the amyloid PET-positive participants, while HC was the most prevalent among amyloid PET-negative participants. Demographics and plasma biomarker values based on clinical diagnoses are presented in Supplementary Table 1.

### Ability of plasma biomarkers to identify amyloid status

**Fig. 1:**
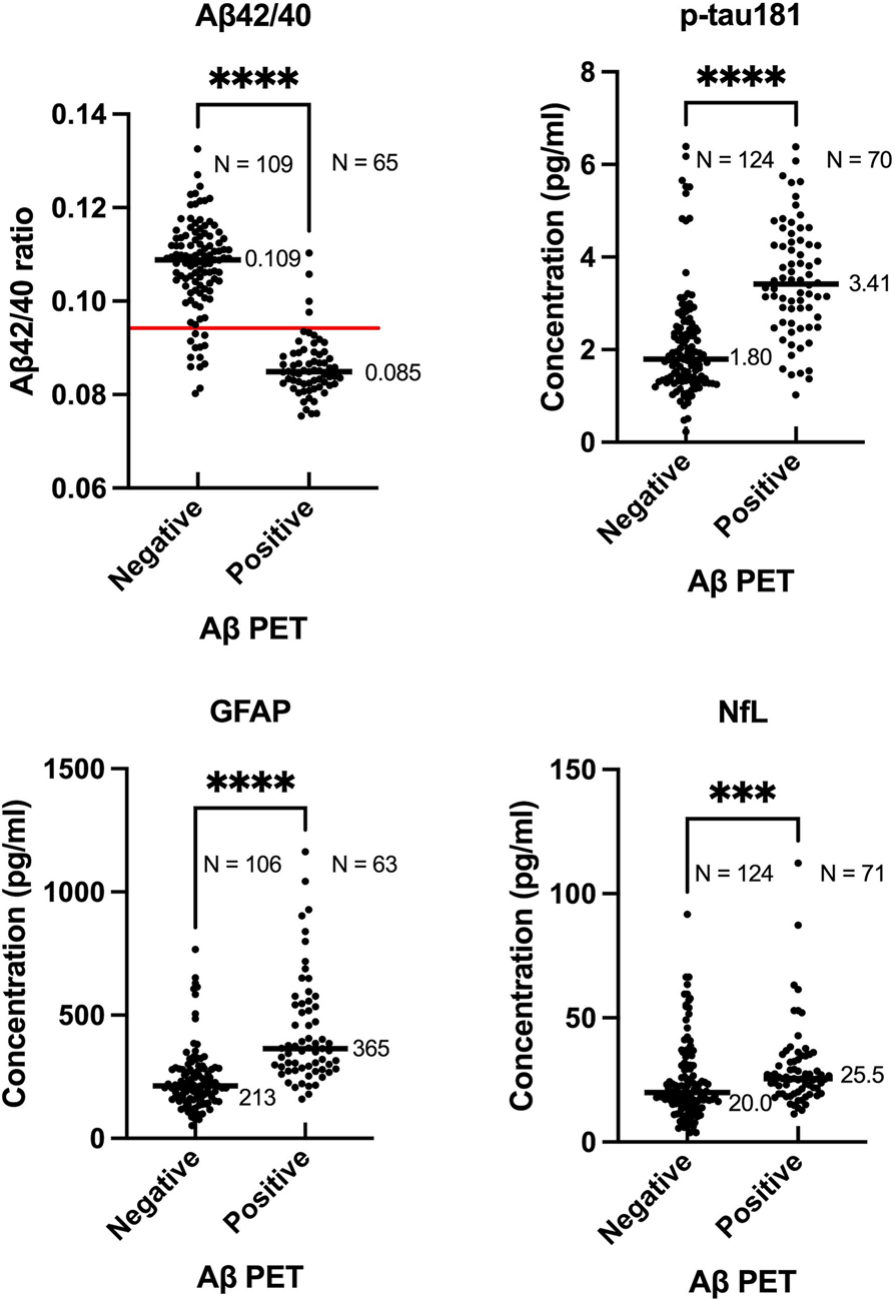
Four plasma biomarkers by visual assessment of amyloid PET. The red horizontal line denotes the Youden-Index-derived best cut-off value (0.0942). Black horizontal lines denote the median concentrations for each plasma biomarker. Differences in plasma biomarker values between Aβ negative and positive participants were analyzed using the Mann–Whitney U test. Abbreviations: Aβ42/40 = amyloid β 42/40 ratio; p-tau181 = tau protein phosphorylated at residue 181; GFAP = glial fibrillary acidic protein; NfL = neurofilament light. *** denotes *p* < 0.001, **** denotes *p* < 0.0001

Figure 1 illustrates the scatterplots of concentrations of the four plasma biomarkers stratified using visually assessed amyloid PET results.

Note that there are slight differences in median values of p-tau181 and NfL between Table 1 and Fig. 1. This discrepancy arises because Table 1 includes only participants who underwent Aβ42/40 measurements, whereas Fig. 1 encompasses all participants with measurements for respective biomarkers, regardless of Aβ42/40 measurement status. Mann–Whitney U tests demonstrated that all biomarkers differed significantly in amyloid PET status. The median values for amyloid PET negative and positive cases were 0.109 and 0.085 for Aβ42/40, 1.80 and 3.41 for p-tau181, 213 and 365 for GFAP, and 20.0 and 25.5 for NfL.

**Fig. 2:**
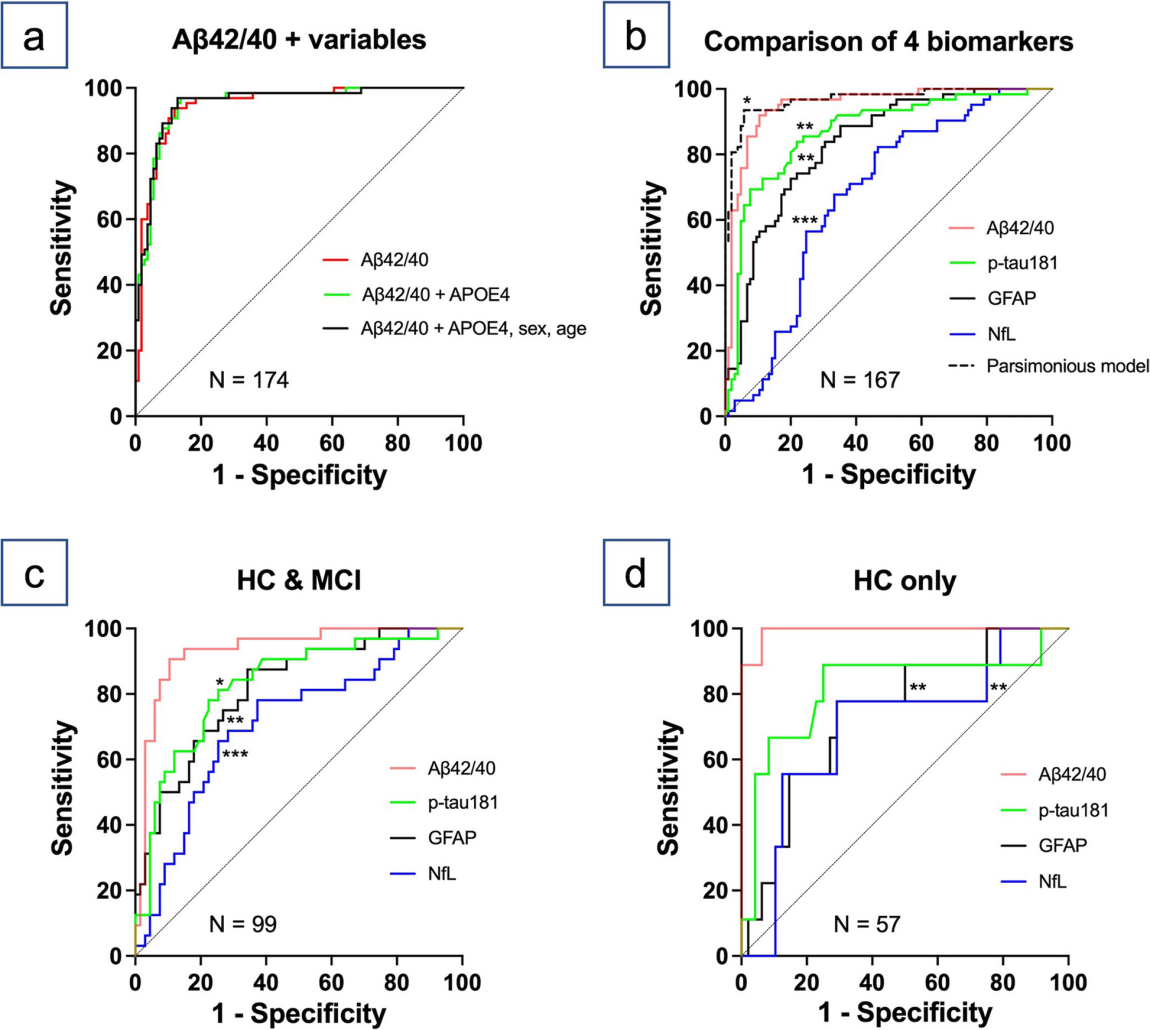
**a–d** ROC curves of Aβ42/40 and other plasma biomarkers. The parsimonious model consists of plasma Aβ42/40, p-tau181, and GFAP. * denotes *p* < 0.05, ** denotes *p* < 0.01, *** denotes *p* < 0.001. Abbreviations: ROC = receiver operating characteristic; Aβ42/40 = amyloid β 42/40 ratio; p-tau181 = tau protein phosphorylated at residue 181; GFAP = glial fibrillary acidic protein; NfL = neurofilament light; HC = healthy control; MCI = mild cognitive impairment

Figure 2a displays the ROC curves for plasma Aβ42/40 using 174 participants with corresponding measurements.

The AUC for Aβ42/40 alone was 0.949. The maximized Youden Index from the ROC curve of Aβ42/40 alone identified the optimal cut-off value as 0.0942, with sensitivity, specificity, positive predictive value, and negative predictive value of 93.9%, 88.1%, 82.4%, and 96%, respectively. Adding APOE4 information yielded an AUC of 0.951, and adding APOE4, sex, and age yielded 0.952. The changes in AUC were insignificant in both models. The forward stepwise logistic regression analysis using 167 participants selected three independent variables (parsimonious model): Aβ42/40, p-tau181, and GFAP. Other variables, such as APOE4, sex, age, MMSE score, or clinical diagnoses, were not selected. The AUC for the parsimonious model was 0.968 (Fig. 2b), which significantly outperformed Aβ42/40 alone (95% confidence interval [CI] for the AUC difference: 0.003–0.033) within the same 167 participants. Figure 2b also presents the comparison of the ROC curves for the four plasma biomarkers. Aβ42/40 exhibited the highest AUC of 0.950, followed by p-tau181 (0.870), GFAP (0.834), and NfL (0.679). The AUC for Aβ42/40 was significantly superior to those for p-tau181 (95%CI: 0.020–0.139), GFAP (95%CI: 0.053–0.179), and NfL (95%CI: 0.182–0.360). Figure 2c and d show the ROC curves of the four biomarkers in 99 HCs and those with MCI and 57 HCs only, respectively. Aβ42/40 significantly outperformed other biomarkers except for p-tau181 in HC only. Table 2 summarizes the AUCs for the four plasma biomarkers.

**Table 2:** The AUCs for the four plasma biomarkers

	*AUC (confidence interval)*					
	*All*		*HC* + *MCI*		*HC only*	
	*N* = *167*	*p-value*	*N* = *99*	*p-value*	*N* = *57*	*p-value*
Aβ42/40	0.950 (0.917–0.983)		0.934 (0.882–0.987)		0.993 (0.977–1.009)	
p-tau181	0.870 (0.812–0.929)	0.009	0.829 (0.741–0.918)	0.023	0.818 (0.623–1.014)	0.080
GFAP	0.834 (0.773–0.895)	0.001	0.818 (0.731–0.905)	0.009	0.745 (0.573–0.918)	0.006
NfL	0.679 (0.597–0.760)	< 0.001	0.711 (0.601–0.821)	< 0.001	0.701 (0.502–0.901)	0.003

*P*-values designate the AUC difference from Aβ42/40 based on DeLong et al. [29]

*Abbreviations*: *AUC* area under the curve, *Aβ42/40* amyloid β 42/40 ratio, *p-tau181* tau protein phosphorylated at residue 181, *GFAP* glial fibrillary acidic protein, *NfL* neurofilament light, *HC* healthy control, *MCI* mild cognitive impairment

### The CL scale and its association with plasma Aβ42/40

Supplementary Fig. 1 shows the CL values for participants categorized based on visual assessment of amyloid PET.

Among the 197 participants who underwent both amyloid PET and one or more plasma biomarker measurements, the calculation of CL for two participants was unable to perform owing to partially corrupted amyloid PET data. The median CL values for the remaining 195 participants were 90.8 for amyloid PET-positive and -2.1 for amyloid PET-negative cases.

**Fig. 3:**
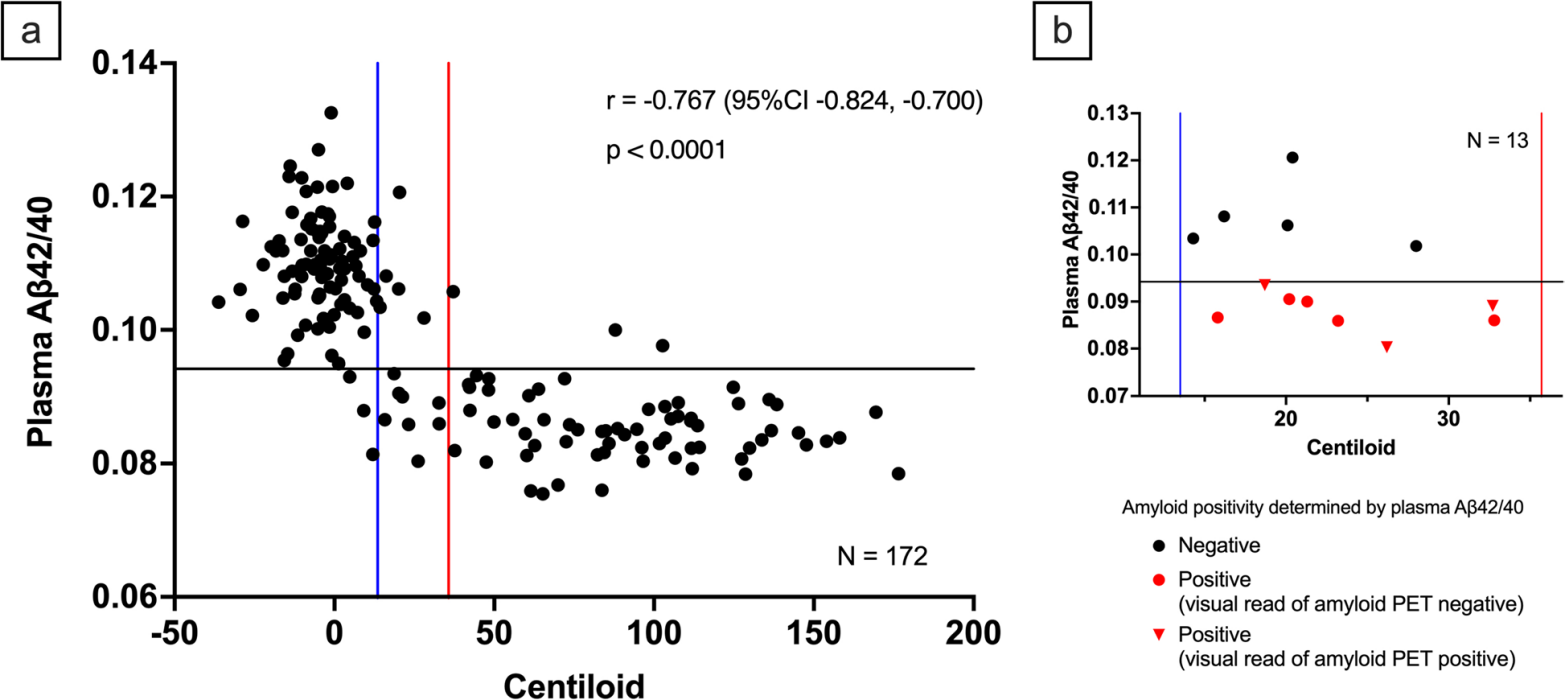
The association between Centiloid and plasma Aβ42/40. **a** represents a scatterplot illustrating the relationship between CL and Aβ42/40. The vertical red and blue lines denote the established (CL = 35.7) and initial (CL = 13.5) amyloid pathology. The black horizontal line indicates the cut-off (0.0942) for plasma Aβ42/40. r denotes Spearman’s rank correlation coefficient. **b** is an enlarged view of the 'gray zone' within Fig. 3a. The black and red colors represent amyloid-negative and -positive determinations by plasma Aβ42/40, respectively. Circles and triangles represent amyloid-negative and -positive determinations by visual assessment of amyloid PET, respectively. Abbreviations: Aβ42/40 = amyloid β 42/40 ratio; CI = confidence interval, CL = Centiloid; PET = positron emission tomography

Figure 3a shows the association between CL and plasma Aβ42/40 for the 172 participants with both data.

The Spearman’s rank correlation coefficient was -0.767 (95%CI -0.924, -0.700, *p* < 0.0001). Figure 3b presents the enlarged image of Fig. 3a between the established (the higher CL cut-off of 35.7) and initial (the lower CL cut-off of 13.5) amyloid pathology (“gray zone” [30]). For participants with CL values between the “gray zone”, 61.5% (8/13 cases) were considered Aβ positive using the cut-off value of 0.0942 for plasma Aβ42/40, as determined by the maximized Youden Index derived from the visual read of amyloid PET as the ground truth. In contrast, 20% (3/15 cases, including two cases without plasma Aβ42/40 measurement) were deemed positive based on the visual assessment of amyloid PET.

Supplementary Table 2 illustrates the ability of plasma Aβ42/40, using the cut-off value of 0.0942, to predict established and initial amyloid pathology as well as visual assessment of amyloid PET.

The sensitivity, specificity, positive predictive value, and negative predictive value were 95.5%, 89.6%, 85.1%, and 96.9%, respectively for established amyloid pathology and 89.9%, 96.8%, 95.9%, and 91.8%, respectively, for initial amyloid pathology. For reference, we also performed the same analyses for other plasma biomarkers. The cut-off value of each plasma biomarker was likewise determined using the maximized Youden Index derived from the visual assessment of amyloid PET as the ground truth. (Supplementary Table 2).

### Correlations between plasma biomarkers, amyloid PET, and cognitive test results

Supplementary Fig. 2 shows the correlations between the four plasma biomarkers, CL, cognitive test results, and age.

Aβ42/40 had the highest correlation with the CL. The correlations of the four biomarkers with cognitive tests were similar, with the absolute values of the coefficients for the MMSE being 0.35–0.41 and for the ADAS-Cog being 0.45–0.49. GFAP had the highest correlation with age. The correlations between plasma Aβ42/40 and amyloid SUVR in the five brain regions (the posterior cingulate cortex and precuneus, frontal cortex, temporal cortex, parietal cortex, and striatum) are presented in Supplementary Fig. 3.

The correlation coefficients were similar to the one between plasma Aβ42/40 and CL (-0.767), ranging from -0.74 to -0.77.

### Correlation between plasma and CSF Aβ42/40

**Fig. 4:**
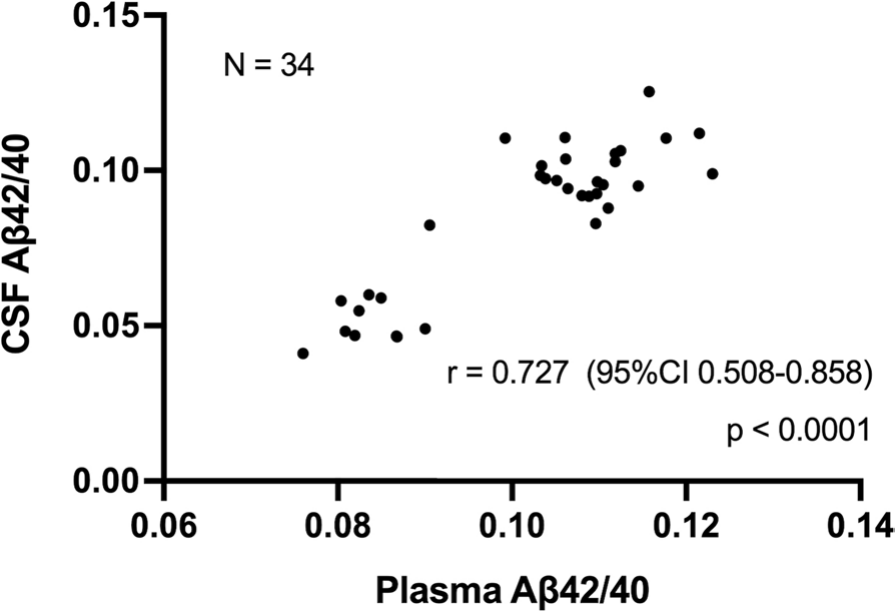
Correlation between plasma and CSF Aβ42/40. Scatterplots showing the correlation between plasma and CSF Aβ42/40. r denotes Spearman's rank correlation coefficient. Abbreviations: CSF = cerebrospinal fluid, Aβ42/40 = amyloid β 42/40 ratio, 95%CI = 95% confidence interval

As shown in Fig. 4, a correlation analysis based on data from 34 participants with available CSF yielded a Spearman's correlation coefficient of 0.727 (95%CI 0.508–0.858, *p* < 0.0001).

## Discussion

This study demonstrated that plasma Aβ42/40 measured using the fully automated HISCL immunoassay in a memory clinic cohort yielded excellent discriminant ability for visually assessed amyloid PET positivity, with an AUC of 0.949. Although adding APOE4, age, and sex did not improve the AUC substantially, the parsimonious model, consisting of plasma p-tau181 and GFAP in addition to Aβ42/40, demonstrated superior performance compared to Aβ42/40 alone. Plasma Aβ42/40 outperformed other plasma biomarkers, including p-tau181, NfL, and GFAP, in the whole and HC plus MCI cohorts. Plasma Aβ42/40 was highly correlated with CL scale and CSF Aβ42/40.

Early screening for AD is crucial for timely intervention, including disease-modifying therapy. As obtaining CSF or amyloid PET is costly and invasive, a safe and easy screening method is desired. Blood-based biomarkers are promising candidates, and numerous related studies have been published. Among the promising candidates is the plasma Aβ42/40 ratio. However, plasma measurements of Aβ were unreliable until recently [5]. One presumed cause is that plasma Aβ42/40 has only a small-fold difference of 11–20% [6, 8, 32] between Aβ-positive and -negative. Thus, as Rave et al. reported, [33] this small-fold difference leads to lower allowable errors in pre-analytic preparation or measurement procedures compared to CSF Aβ42/Aβ40 and plasma p-tau. Consequently, standard plate-based enzyme-linked immunosorbent assay methods, which have a relatively high intra-assay coefficient of variation (6–24% for Aβ42 and 8–14% for Aβ40 [34]), would not accurately measure the small difference. In contrast, findings using immunoprecipitation-mass spectrometry (IP-MS) were encouraging. A recent review [35] reported that IP-MS-based measurements yielded an excellent weighted average AUC (0.866 using CSF as a reference and 0.834 using PET as a reference). In contrast, the chemiluminescence assay (AUC of 0.803 and 0.818, respectively) or Simoa-based assay (AUC of 0.726 and 0.690) yielded inferior results. A head-to-head comparison study of eight plasma Aβ42/40 assays also reported superior results using MS-based methods [36] compared with immunoassay platforms. Nonetheless, their high cost and complex procedures may hinder widespread use. Compared with IP-MS, immunoassay platforms are easier to implement with lower costs. Lately, fully automated immunoassay platforms have reported favorable results. For example, the Elecsys immunoassay reported AUCs of 0.83–0.87 [6]. Moreover, the HISCL immunoassay achieved AUCs of 0.87–0.94 [8] and highly correlated with IP-MS results [7]. The strengths of the fully automated equipment include more exact measurements without manual steps compared with traditional procedures, such as enzyme-linked immunosorbent assay. The coefficients of variations obtained using the HISCL assay were much better (2.0–3.7% for Aβ42 and 1.7–2.0% for Aβ40) [7] than those obtained using traditional immunoassays. Furthermore, because they are equipment already on the market, they have the potential for large-scale clinical application as a screening tool. However, their utility must be confirmed across multiple independent cohorts with more diverse participants from different areas or regions before clinical implementation. Furthermore, it is imperative to examine their ability to detect early amyloid pathology.

In this study, we examined the utility of the HISCL immunoassay with a well-characterized memory clinic cohort. The original HISCL study [8] only included participants with AD continuum from a clinical trial population; in contrast, our cohort encompassed various clinical patients [9] with diagnoses of FTLD, DLB/PD, psychiatric disorders, and TBI. Here, employing the visual read of amyloid PET as a gold standard, we confirmed the excellent discriminant ability of plasma Aβ42/40 measured using the HISCL platform, although the best cut-off values were slightly different, with 0.0942 in the present study versus 0.102 in the original study [8]. The HISCL platform achieved a sensitivity of 93.9% with only four false negatives; however, its specificity was lower (88.1%) with 13 false positives, which may be explained by the hypothesis that plasma Aβ42/40 declines before amyloid PET turns positive [32]. This hypothesis was also supported by the association with CL. When the CL cut-off was set at 35.7 for established amyloid pathology, its specificity was 89.6%. However, it improved to 96.8% when the cut-off was set at 13.5 for initial amyloid pathology. The results suggest that the HISCL assay can detect the “gray zone” that may indicate future Aβ accumulation [30]. In “gray zone” cases, plasma Aβ42/40 compared favorably with the visual read of amyloid PET in determining Aβ positivity (61.5% for plasma Aβ42/40 vs. 20% for amyloid PET). Thus, these results highlight the potential of the HISCL assay to detect challenging cases with a subtle accumulation of brain amyloid.

In our study, plasma Aβ42/40 outperformed other Simoa-based plasma biomarkers, such as p-tau181, NfL, or GFAP, in differentiating visually assessed amyloid PET positivity. Plasma p-tau181 is one of the most studied plasma biomarkers [37] and has been reported to detect Aβ accumulation in the brain [38, 39]. A study reported that Simoa-based plasma p-tau181 was superior to the same Simoa-based plasma Aβ42/40 [10]. In contrast, its measurement using the HISCL platform performed significantly better than Simoa-based p-tau181 in our study. NfL is a generic marker of neurodegeneration and may not be specific to amyloid pathological changes [37], which explains the relatively low AUC. However, GFAP, a marker of astrocyte reactivity, can detect early Aβ accumulation [40, 41]. Notably, Aβ42/40 surpassed GFAP when using the whole sample population and the HCs plus participants with MCI or HCs only. The results imply that plasma Aβ42/40 measured using the HISCL platform may be a useful screening tool as an early marker of amyloid pathology. Moreover, the incorporation of supplementary measurements of plasma p-tau181 and GFAP to Aβ42/40 exhibited enhanced predictive ability compared to Aβ42/40 measurement only. Despite the increased costs, the combination of these measurements may serve as a more proficient screening tool than measuring Aβ42/40 alone or be beneficial for diagnostically challenging cases.

Plasma Aβ42/40 was highly correlated with CSF Aβ42/40 (Fig. 4). In a recent study of a head-to-head comparison of eight plasma Aβ42/40 assays, Spearman’s correlations ranged from 0.147 to 0.655 [36]. Despite the small sample size of 34, our result of 0.727 was superior, illustrating the reliability of the HISCL assay.

In addition to using the fully automated immunoassay platform, one of the strengths of our study was our rigorous plasma preparation protocol. Besides being adherent to the manufacturer’s reference manual, we followed additional steps such as placing plasma in iced water, centrifuging it twice within 2 h of extraction, and storing them at -80°C. These measures may have contributed to the high accuracy of our results. Future research should investigate which steps of plasma preparation are critical for achieving high accuracy.

### Limitations

This study has some limitations. The participants encompassed a diverse patient population; however, this is a study from a single center in Japan, and thus they were racially and ethnically homogenous. However, the original study included racially diverse participants. Second, studies have reported that plasma tau phosphorylated at residue 217 (p-tau217) or residue 231 (p-tau231) may be more effective in distinguishing amyloid pathology than Aβ42/40 [42, 43]. Future studies should compare the utility of Aβ42/40 using the HISCL platform with these plasma phospho-tau assays. Third, we did not measure plasma Aβ42/40 using alternative assays to compare with the HISCL platform in distinguishing amyloid positivity.

## Conclusion

Our study demonstrated the utility of plasma Aβ42/40 measured using the fully automated HISCL immunoassay platform in predicting PET-derived Aβ positivity in a diverse patient population and healthy controls. It has the potential to identify early amyloid pathology. Furthermore, the equipment is widely used in clinical settings; hence, it may be used as a large-scale screening tool for the incoming era of AD-modifying therapies.

### Supplementary Information

**Additional file 1: Supplementary Table 1.** Demographics and plasma biomarker values based on clinical diagnoses.

**Additional file 2: Supplementary Fig. 1.** Centiloid values based on the visual assessment of amyloid PET. **** denotes *p* < 0.0001. The red and blue horizontal bars denote the higher and lower CL cut-offs, respectively. The solid black horizontal lines denote the median CL values. Differences in CL values between visual-read Aβ negative and positive participants were analyzed using the Mann–Whitney U test. Abbreviations: Aβ = amyloid β.

**Additional file 3: Supplementary Table 2.** The performances of plasma biomarkers in predicting the amyloid pathologies defined by visual assessment and CL scale.

**Additional file 4: Supplementary Fig. 2.** Correlations between the four plasma biomarkers, Centiloid, cognitive test results, and age. Heat map showing the correlations between the four plasma biomarkers, Centiloid, cognitive test results, and age. The number in each cell represents Spearman’s rank correlation coefficient between the items in the corresponding row and column. Abbreviations: Aβ42/40 = amyloid β 42/40 ratio; p-tau181 = tau protein phosphorylated at residue 181; NfL = neurofilament light, GFAP = glial fibrillary acidic protein; MMSE = Mini-Mental State Examination; ADAS-Cog = Alzheimer’s Disease Assessment Scale Cognitive Behavior Section.

**Additional file 5: Supplementary Fig. 3.** Scatterplots showing the correlations between plasma Aβ42/40 and amyloid SUVR in the frontal, parietal, and temporal cortices and posterior cingulate gyrus and precuneus, and striatum. r denotes Spearman's rank correlation coefficient. Abbreviations: SUVR = standard uptake value ratio, Aβ42/40 = amyloid β 42/40 ratio, 95%CI = 95% confidence interval.

## Data Availability

The datasets used and analyzed during the current study will be available from the corresponding author upon reasonable request.

## Abbreviations

ADAS-cog: The Alzheimer’s Disease Assessment Scale Cognitive Behavior Section
Aβ: Amyloid β
Aβ42/40: Amyloid β 42/40 ratio
APOE: Apolipoprotein E
AUC: Area under the curve
CDR: The Clinical Dementia Rating
CI: Confidence interval
CL: Centiloid
CSF: Cerebrospinal fluid
DLB/PD: Dementia with Lewy bodies/Parkinson’s disease
DNA: Deoxyribonucleic acid
GFAP: Glial fibrillary acidic protein
FTLD: Frontotemporal lobar degeneration
HC: Healthy controls
HISCL: High-sensitivity chemiluminescence enzyme
MCI: Mild cognitive impairment
MRI: Magnetic resonance imaging
MMSE: Mini-Mental State Examination
NfL: Neurofilament light
PET: Positron emission tomography
p-tau181: Phosphorylated tau at residue 181
RCTU: Regional cortical tracer uptake
ROC: Receiver operating characteristic
