# Supplementary material for: Performance of plasma Aβ42/40, measured using a fully automated immunoassay, across a broad patient population in identifying amyloid status
Source: Alzheimers Res Ther. 2023 Sep 4;15:149. doi: 10.1186/s13195-023-01296-5 (PMC10476307; doi:10.1186/s13195-023-01296-5)
Supplement: Supplementary file 1 — Additional file 1: Supplementary Table 1. Demographics and plasma biomarker values based on clinical diagnoses. [file 13195_2023_1296_MOESM1_ESM.docx]

Supplementary Table 1. Demographics and plasma biomarker values based on clinical diagnoses

|  |  | *Mean (SD) or N* | | | | | |
| --- | --- | --- | --- | --- | --- | --- | --- |
|  | *Total Measurements* | *HC* | *MCI* | *AD* | *FTLD* | *DLB/PD* | *Others* |
|  |  | *63* | *50* | *38* | *27* | *5* | *14* |
| Sex, No. of male and female  participants | 197 | 33/30 | 30/20 | 17/21 | 11/16 | 2/3 | 7/7 |
| Age | 197 | 70.0  (7.7) | 72.7  (9.7) | 72.9  (10.3) | 65.8  (10.3) | 63.8  (11.9) | 57.2  (13.8) |
| APOE ɛ4, No. of positive and negative participants | 192 | 17/45 | 19/30 | 19/16 | 7/20 | 2/3 | 4/10 |
| MMSE score | 194 | 28.9  (1.1) | 27.0  (2.1) | 19.8  (4.3) | 22.3  (8.4) | 24.2  (8.6) | 28.4  (1.7) |
| ADAS-Cog score | 193 | 4.1  (2.2) | 7.9  (3.8) | 17.7  (8.6) | 16.6  (15.7) | 11.4  (14.6) | 3.9  (1.9) |
| Aβ42/40 | 174 | 0.105  (0.013) | 0.094  (0.012) | 0.088  (0.010) | 0.104  (0.012) | 0.101  (0.010) | 0.107  (0.009) |
| p-tau181 (pg/ml) | 194 | 2.03  (1.07) | 3.00  (1.33) | 3.41  (1.17) | 2.62  (1.68) | 2.46  (0.57) | 1.48  (0.65) |
| GFAP (pg/ml) | 169 | 238  (99) | 362  (207) | 380  (125) | 377  (312) | 285  (191) | 207  (159) |
| NfL (pg/ml) | 195 | 19.6  (9.9) | 28.1  (13.6) | 26.7  (9.8) | 42.5  (22.9) | 24.9  (16.5) | 19.6  (22.4) |
| Abbreviations: SD = standard deviation; APOE = apolipoprotein E; MMSE = Mini-Mental State Examination; ADAS-Cog = Alzheimer’s Disease Assessment Scale Cognitive Behavior Section; Aβ42/40 = amyloid β 42/40 ratio; p-tau181 = tau protein phosphorylated at residue 181; NfL = neurofilament light, GFAP = glial fibrillary acidic protein; HC = healthy control; MCI = mild cognitive impairment; AD = Alzheimer’s disease; FTLD = frontotemporal lobar degeneration; DLB/PD = dementia with Lewy bodies/Parkinson’s disease. | | | | | | | |
