# Supplementary figures and images for: Performance of plasma Aβ42/40, measured using a fully automated immunoassay, across a broad patient population in identifying amyloid status
Source: Alzheimers Res Ther. 2023 Sep 4;15:149. doi: 10.1186/s13195-023-01296-5 (PMC10476307; doi:10.1186/s13195-023-01296-5)

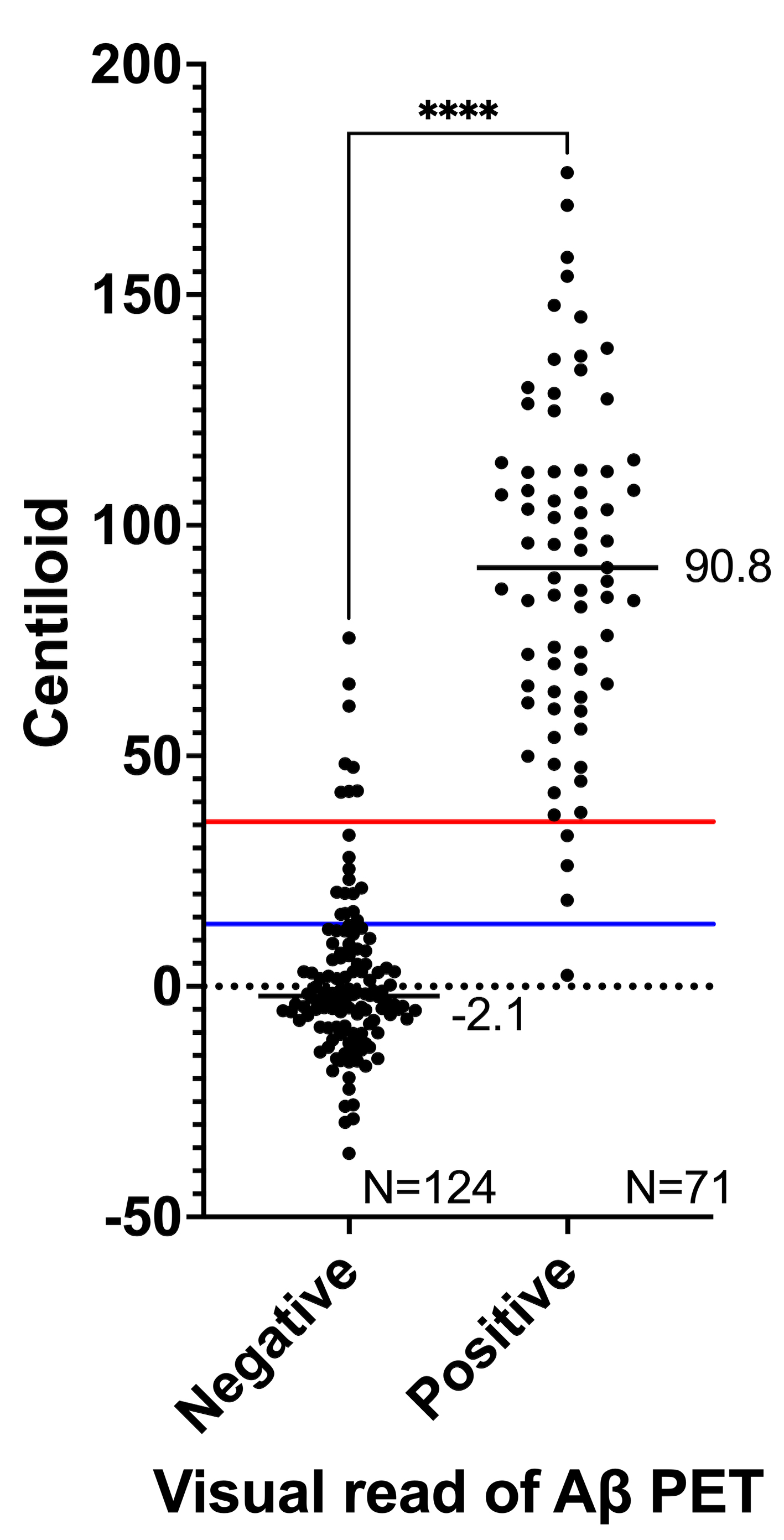

Supplement: Supplementary file 2 — Additional file 2: Supplementary Fig. 1. Centiloid values based on the visual assessment of amyloid PET. **** denotes p < 0.0001. The red and blue horizontal bars denote the higher and lower CL cut-offs, respectively. The solid black horizontal lines denote the median CL values. Differences in CL values between visual-read Aβ negative and positive participants were analyzed using the Mann–Whitney U test. Abbreviations: Aβ = amyloid β. [file 13195_2023_1296_MOESM2_ESM.jpg]

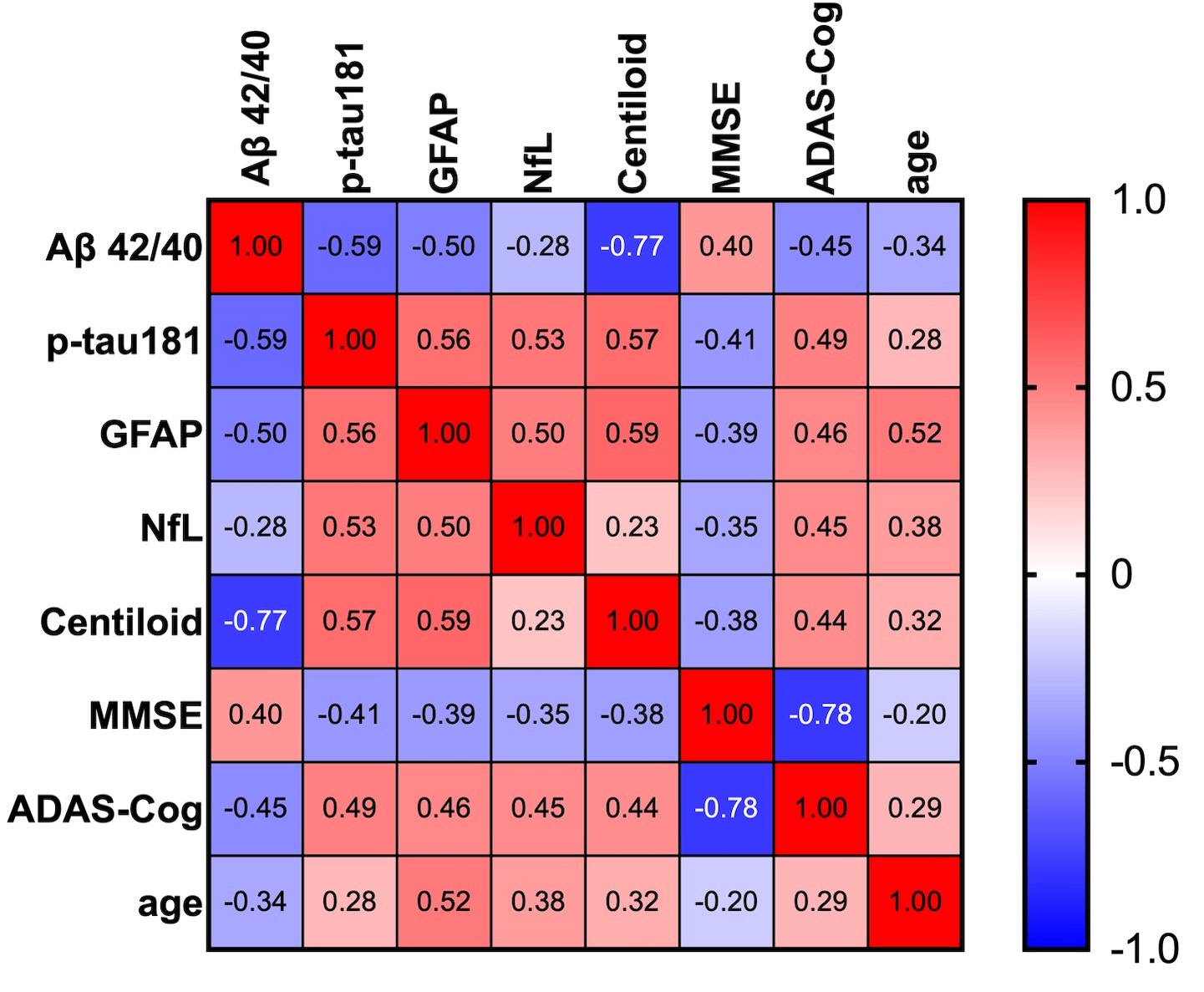

Supplement: Supplementary file 4 — Additional file 4: Supplementary Fig. 2. Correlations between the four plasma biomarkers, Centiloid, cognitive test results, and age. Heat map showing the correlations between the four plasma biomarkers, Centiloid, cognitive test results, and age. The number in each cell represents Spearman’s rank correlation coefficient between the items in the corresponding row and column. Abbreviations: Aβ42/40 = amyloid β 42/40 ratio; p-tau181 = tau protein phosphorylated at residue 181; NfL = neurofilament light, GFAP = glial fibrillary acidic protein; MMSE = Mini-Mental State Examination; ADAS-Cog = Alzheimer’s Disease Assessment Scale Cognitive Behavior Section. [file 13195_2023_1296_MOESM4_ESM.jpg]

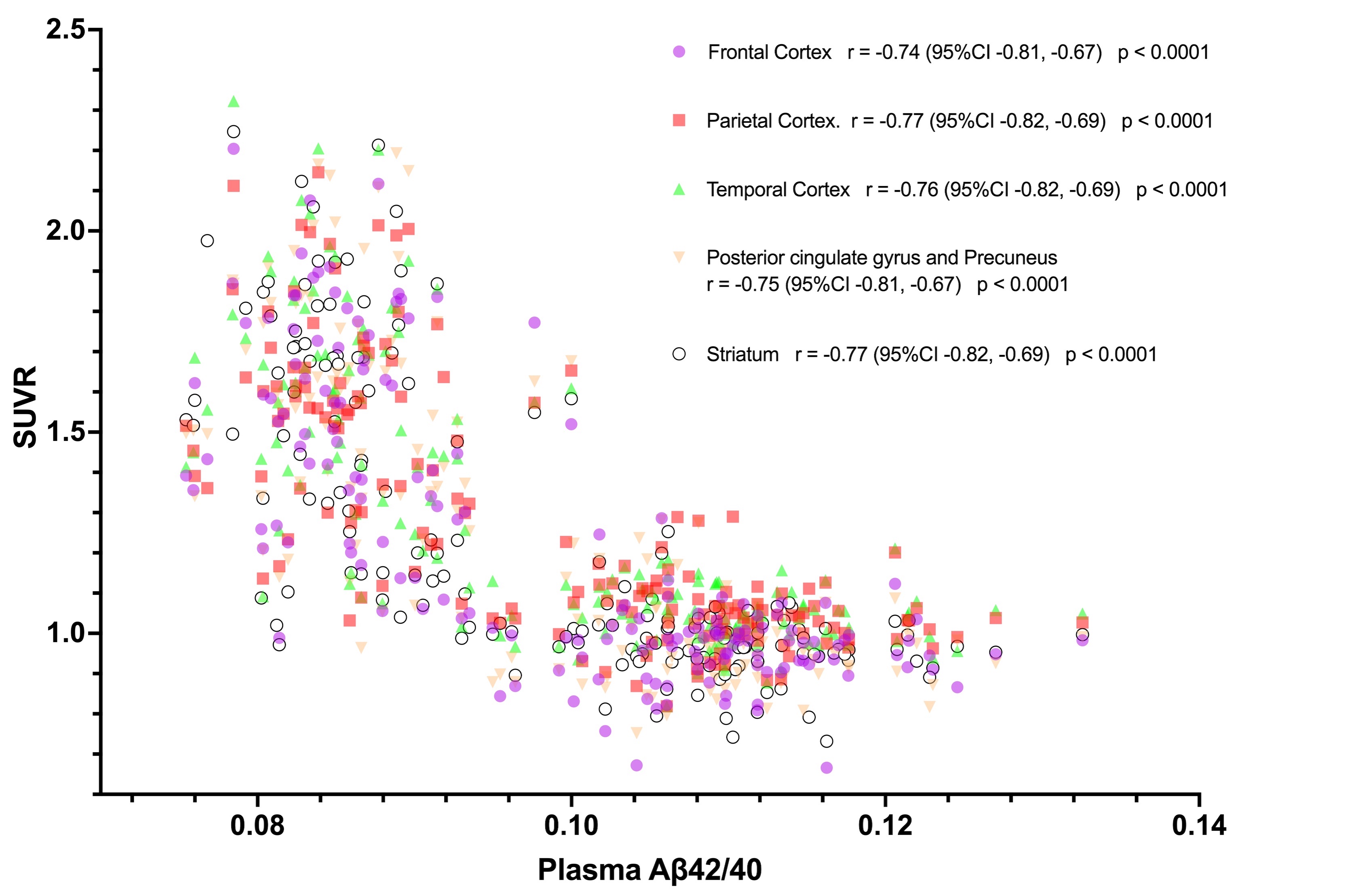

Supplement: Supplementary file 5 — Additional file 5: Supplementary Fig. 3. Scatterplots showing the correlations between plasma Aβ42/40 and amyloid SUVR in the frontal, parietal, and temporal cortices and posterior cingulate gyrus and precuneus, and striatum. r denotes Spearman's rank correlation coefficient. Abbreviations: SUVR = standard uptake value ratio, Aβ42/40 = amyloid β 42/40 ratio, 95%CI = 95% confidence interval. [file 13195_2023_1296_MOESM5_ESM.jpg]
