# Supplementary material for: Performance of plasma Aβ42/40, measured using a fully automated immunoassay, across a broad patient population in identifying amyloid status
Source: Alzheimers Res Ther. 2023 Sep 4;15:149. doi: 10.1186/s13195-023-01296-5 (PMC10476307; doi:10.1186/s13195-023-01296-5)
Supplement: Supplementary file 3 — Additional file 3: Supplementary Table 2. The performances of plasma biomarkers in predicting the amyloid pathologies defined by visual assessment and CL scale. [file 13195_2023_1296_MOESM3_ESM.docx]

Supplementary Table 2. The performances of plasma biomarkers in predicting the amyloid pathologies defined by visual assessment and CL scale

|  |  | Sensitivity | Specificity | PPV | NPV |
| --- | --- | --- | --- | --- | --- |
| Aβ42/40  (cut-off = 0.0942) | Visual assessment | 93.9% | 88.1% | 82.4% | 96% |
|  | Established pathology | 95.5% | 89.6% | 85.1% | 96.9% |
|  | Initial pathology | 89.9% | 96.8% | 95.9% | 91.8% |
| p-tau181  (cut-off = 2.88) | Visual assessment | 72.9% | 85.5% | 73.9% | 84.8% |
|  | Established pathology | 74.0% | 87.5% | 78.3% | 84.7% |
|  | Initial pathology | 64.8% | 88.6% | 82.6% | 75.0% |
| GFAP  (cut-off = 248) | Visual assessment | 88.9% | 64.2% | 59.6% | 90.7% |
|  | Established pathology | 85.9% | 63.1% | 59.1% | 87.8% |
|  | Initial pathology | 81.8% | 66.7% | 67.7% | 81.1% |
| NfL  (cut-off = 18.0) | Visual assessment | 88.7% | 41.1% | 46.3% | 86.4% |
|  | Established pathology | 86.7% | 40.3% | 47.8% | 82.8% |
|  | Initial pathology | 83.3% | 41.3% | 55.1% | 74.1% |
| The cut-off value of each plasma biomarker was determined using the maximized Youden Index derived from the visual assessment of the amyloid PET as the ground truth.  The established and initial pathologies were defined using CL cut-offs of 35.7 and 13.5, respectively.  Abbreviations: CL = Centiloid; PPV = positive predictive value; NPV = negative predictive value; Aβ42/40 = amyloid β 42/40 ratio; p-tau181 = tau protein phosphorylated at residue 181; GFAP = glial fibrillary acidic protein; NfL = neurofilament light. | | | | | |
